# Susceptibility of Gram-negative pathogens collected in Israel to ceftolozane/tazobactam, imipenem/relebactam and comparators: SMART 2018–22

**DOI:** 10.1093/jacamr/dlae150

**Published:** 2024-09-17

**Authors:** Mark G Wise, C Andrew DeRyke, Irina Alekseeva, Fakhar Siddiqui, Katherine Young, Mary R Motyl, Daniel F Sahm

**Affiliations:** IHMA, Schaumburg, IL, USA; IHMA, Schaumburg, IL, USA; MSD, Dubai, United Arab Emirates; Merck & Co., Inc., Rahway, NJ, USA; Merck & Co., Inc., Rahway, NJ, USA; Merck & Co., Inc., Rahway, NJ, USA; IHMA, Schaumburg, IL, USA

## Abstract

**Objectives:**

To assess the *in vitro* antimicrobial activity of ceftolozane/tazobactam, imipenem/relebactam and comparator agents against clinical isolates of Gram-negative bacilli collected in Israel from 2018 to 2022.

**Methods:**

Six clinical laboratories each collected up to 250 consecutive Gram-negative isolates per year from patients with bloodstream, intra-abdominal, lower respiratory tract and urinary tract infections. MICs were determined by CLSI broth microdilution and interpreted with 2024 EUCAST breakpoints. Acquired β-lactamase gene carriage was investigated for most ceftolozane/tazobactam- and imipenem/relebactam-resistant isolates.

**Results:**

Among the full collection of Enterobacterales (*n *= 4420), 95.1% were susceptible to ceftolozane/tazobactam, including 95.3% of putative AmpC/ESBL-positive, non-carbapenem-resistant Enterobacterales (CRE) phenotype *Escherichia coli* and 86.6% of AmpC/ESBL-positive, non-CRE phenotype *Klebsiella pneumoniae*. Overall, 99.8% of non-*Morganellaceae* Enterobacterales (*n* = 3723) were imipenem/relebactam susceptible including 98% of the MDR isolates. Most *Pseudomonas aeruginosa* isolates (*n *= 1182) were inhibited by ceftolozane/tazobactam (93.9% susceptible) and imipenem/relebactam (94.7%). Imipenem/relebactam retained activity against ≥78% of cefepime-resistant, ceftazidime-resistant, and piperacillin/tazobactam-resistant *P. aeruginosa*, while ceftolozane/tazobactam inhibited the greatest percentage of meropenem-resistant *P. aeruginosa* (67.4%) among comparator β-lactam antimicrobials. Molecular characterization showed the majority of imipenem/relebactam-resistant Enterobacterales harboured a metallo-β-lactamase, while half of the ceftolozane/tazobactam-resistant Enterobacterales carried an acquired ESBL or AmpC. Most of the imipenem/relebactam- and ceftolozane/tazobactam-resistant *P. aeruginosa* characterized did not possess acquired β-lactamases.

**Conclusions:**

Recent clinical isolates of Enterobacterales and *P. aeruginosa* collected in Israel were highly susceptible to ceftolozane/tazobactam and imipenem/relebactam.

## Introduction

Ceftolozane/tazobactam and imipenem/relebactam are newer β-lactam/β-lactamase inhibitor combinations that have been approved by both the FDA and EMA for treatment of serious infections, including hospital-acquired bacterial pneumonia. Routine surveillance of these newer agents is critical in the ongoing effort to define their utility and appropriate use. Surveillance data describing *in vitro* susceptibility testing results for ceftolozane/tazobactam and imipenem/relebactam against clinical isolates of Gram-negative bacilli from Israel have been generally presented as a part of the Middle East region as a whole,^1–3^ and data specific to isolates collected in Israel are sparse. Thus, we evaluated the activity of these two agents and relevant comparators against isolates of Gram-negative bacilli collected by clinical laboratories in Israel as part of the Study for Monitoring Antimicrobial Resistance Trends (SMART) global surveillance programme.

## Materials and methods

### Bacterial isolates and antimicrobial susceptibility testing

From 2018 to 2022, six clinical laboratories in Israel participated in the SMART programme. Each laboratory collected consecutive, aerobic or facultative Gram-negative isolates from intra-abdominal, urinary tract, lower respiratory tract and bloodstream infections. Only one isolate per patient per species per year was accepted. All isolates were sent to a central laboratory (IHMA, Monthey, Switzerland), where species identity was confirmed using MALDI-TOF mass spectrometry (Bruker Daltonics, Billerica, MA, USA) and antimicrobial susceptibility performed following the CLSI reference broth microdilution method.^4^ MICs were interpreted using 2024 EUCAST breakpoints.^5^ EUCAST does not publish breakpoints for imipenem/relebactam against *Morganellaceae* family (*Proteus*, *Providencia* and *Morganella* spp.) because they display intrinsic, lowered susceptibility to imipenem by a mechanism independent of β-lactamase production^6^ and relebactam does not improve the activity of imipenem against *Morganellaceae*. Therefore, imipenem/relebactam susceptibility was analysed for non-*Morganellaceae* Enterobacterales (NME) only.

A putative AmpC/ESBL-positive non-carbapenem-resistant Enterobacterales (CRE) phenotype was defined for isolates of *Escherichia coli* and *Klebsiella pneumoniae* as those testing with a ceftazidime MIC ≥ 2 mg/L and a meropenem MIC ≤ 1 mg/L. A MDR phenotype was defined as resistance (EUCAST) to ≥3 sentinel agents [amikacin, aztreonam, cefepime, ceftazidime (Enterobacterales only), colistin, imipenem, levofloxacin and piperacillin/tazobactam]. Difficult-to-treat resistance (DTR) was defined as non-susceptibility (EUCAST) to all β-lactams (including aztreonam, ceftazidime, cefepime, imipenem, meropenem and piperacillin/tazobactam), as well as fluoroquinolones (levofloxacin).^7^

### Screening for β-lactamase genes

Isolates meeting the following phenotypic criteria were screened for β-lactamase genes: NME isolates (excluding *Serratia* spp.) testing with imipenem or imipenem/relebactam MIC values of ≥2 mg/L and *Pseudomonas aeruginosa* isolates testing with imipenem or imipenem/relebactam MIC values of ≥4 mg/L; Enterobacterales and *P. aeruginosa* isolates testing with ceftolozane/tazobactam MIC values of ≥4 and ≥8 mg/L, respectively. Published multiplex PCR assays were used to screen for β-lactamase genes as described previously.^8,9^ For *P. aeruginosa* collected in 2020–22 only, isolates were characterized by short-read whole-genome sequencing (Illumina Hiseq 2 × 150-bp reads) to a targeted coverage depth of 100×^10^ and analysed using the CLC Genomics Workbench (Qiagen). The ResFinder database was used to detect β-lactamase genes.^11^ In total, 214 NME were molecularly characterized. Per SMART protocol for *P. aeruginosa*, a representative sample of approximately 75% of isolates meeting the criteria for molecular characterization was characterized for β-lactamase genes (54 randomly selected isolates of 293 that qualified were not characterized).

## Results

A brief summary of the demographic and clinical characteristics associated with all isolates of Enterobacterales and *P. aeruginosa* collected in Israel in 2018–22 is provided in Table S1 (available as Supplementary data at *JAC-AMR* Online).

Ceftolozane/tazobactam inhibited 95.1% of all Enterobacterales isolates, including 98.4% of *E. coli* isolates, 91.8% of *K. pneumoniae* isolates, 100% of *Klebsiella oxytoca* isolates, 94.5% of *Citrobacter* spp. and 100% of *Serratia* spp. (Table 1). Reduced ceftolozane/tazobactam activity was observed against organisms with intrinsic AmpC-type enzymes, such as *Enterobacter* spp. (79.6% susceptible) and *Klebsiella aerogenes* (77.2% susceptible). Regarding *E. coli* and *K. pneumoniae* exhibiting the putative AmpC/ESBL-positive, non-CRE phenotype, 95.3% and 86.6% were inhibited by ceftolozane/tazobactam, respectively. 80.4% of the MDR Enterobacterales isolates were susceptible to ceftolozane/tazobactam; however, none of the 14 DTR isolates were susceptible. Imipenem/relebactam was highly active, inhibiting 99.8% of NME and 100% of AmpC/ESBL-positive, non-CRE phenotype *E. coli* and *K. pneumoniae*. Meropenem (99.3% susceptible), ceftazidime/avibactam (99.7%) and amikacin (97.7%) also inhibited very high percentages of Enterobacterales isolates, while cefepime, ceftazidime, piperacillin/tazobactam and levofloxacin were less active, inhibiting <87% of the isolates. Colistin inhibited 81.6% of all Enterobacterales, but 92.3% of the NME, as it is inactive against *Morganellaceae* species.^12^ Both imipenem/relebactam (98.0% susceptible) and ceftazidime/avibactam (98.9%) demonstrated potent activity versus Enterobacterales with an MDR phenotype, and each inhibited 11/14 (78.6%) of DTR isolates.

**Table 1.** Antimicrobial susceptibility of clinical isolates of Enterobacterales and *P. aeruginosa* collected in Israel 2018–22

		% Susceptible										
Organism group or phenotype	*n*	C/T	IMR	IPM^a,b^	MEM	CZA	CAZ^b^	FEP^b^	TZP^b^	LVX^b,c^	AMK	CST
Enterobacterales	4420	95.1	NA	97.8	99.3	99.7	70.9	73.6	86.9	68.4	97.7	81.6
NME	3723	94.7	99.8	99.3	99.2	99.8	69.2	73.7	85.5	71.6	98.0	92.3
*E. coli*	1883	98.4	99.9	99.8	99.8	99.8	70.6	73.7	91.3	64.8	97.9	99.4
ESBL non-CRE^d^	549	95.3	100	100	100	100	0.0	14.8	77.8	29.9	94.5	98.7
*K. pneumoniae*	923	91.8	99.6	98.2	97.8	99.6	54.6	56.6	75.8	67.2	97.4	99.2
ESBL non-CRE^d^	396	86.6	100	100	100	100	0.0	7.1	56.1	40.2	96.5	99.5
*Citrobacter* spp.	183	94.5	100	99.5	99.5	100	86.3	94.5	89.1	92.9	100	100
*Enterobacter* spp.	226	79.6	100	99.6	99.6	100	66.8	79.2	74.8	86.3	99.1	82.7
*K. aerogenes*	123	77.2	100	100	100	100	54.5	90.2	57.7	87.8	100	100
*K. oxytoca*	100	95.0	98.0	98.0	99.0	98.0	91.0	95.0	92.0	95.0	98.0	99.0
*Serratia* spp.	231	100	100	100	100	100	99.6	99.1	94.4	90.9	98.3	2.6
MDR	998	80.4	98.0	95.6	97.0	98.9	6.6	9.5	56.5	28.9	91.7	85.8
DTR	14	0.0	78.6	0.0	0.0	78.6	0.0	0.0	0.0	0.0	35.7	92.9
*P. aeruginosa*	1182	93.9	94.7	82.4	82.3	95.2	77.2	81.7	76.4	75.9	95.6	99.5
FEP-resistant	216	68.1	78.2	53.2	50.0	75.0	11.6	0	12.0	37.0	81.0	99.1
CAZ-resistant	269	74.0	82.9	61.0	58.0	79.2	0	29.0	13.0	48.0	84.4	99.3
MEM-resistant	92	67.4	44.6	2.2	0	59.8	32.6	33.7	19.6	35.9	76.1	100
TZP-resistant	279	77.4	81.4	58.8	54.8	80.3	16.1	31.9	0	48.0	86.7	99.6
MDR	240	71.7	75.8	44.2	41.7	77.1	16.7	16.7	10.4	32.5	80.8	98.3
DTR	33	39.4	27.3	0	0	21.2	0	0	0	0	57.6	100

AMK, amikacin; C/T, ceftolozane/tazobactam; CAZ, ceftazidime; CRE, carbapenem-resistant Enterobacterales; CST, colistin; CZA, ceftazidime/avibactam; DTR, difficult-to-treat resistant; ESBL, extended-spectrum β-lactamase; FEP, cefepime; IMR, imipenem/relebactam; IPM, imipenem; LVX, levofloxacin; MDR, multi-drug resistant; MEM, meropenem; NA, not applicable or MIC breakpoint not available; NME, non-*Morganellaceae* Enterobacterales; TZP, piperacillin/tazobactam.

^a^The results combine % susceptible, increased exposure values for *Morganellaceae* and % susceptible values for non-*Morganellaceae* Enterobacterales.

^b^For IPM, CAZ, FEP, TZP and LVX against *P. aeruginosa*, the results represent ‘% susceptible, increased exposure’ as defined in the EUCAST guidelines.

^c^Levofloxacin was only tested against Enterobacterales isolates from 2018 to 2021.

^d^Putative ESBL non-CRE phenotype was defined by an isolate testing with a CAZ MIC ≥ 2 mg/L and a MEM MIC ≤ 1 mg/L.

Ceftolozane/tazobactam and imipenem/relebactam were both active against the full collection of *P. aeruginosa* isolates (*n *= 1182), with 93.9% and 94.7 of the population susceptible, respectively (Table 1). Ceftolozane/tazobactam and imipenem/relebactam retained activity against 68%–77% (ceftolozane/tazobactam) and 78%–82% (imipenem/relebactam) of cefepime-resistant, ceftazidime-resistant and piperacillin/tazobactam-resistant isolates. Against meropenem-resistant isolates, 67.4% and 44.6% were ceftolozane/tazobactam and imipenem/relebactam susceptible, respectively. Both ceftolozane/tazobactam (71.7%) and imipenem/relebactam (75.8%) inhibited >70% of the MDR *P. aeruginosa,* but <40% of the DTR subset. Ceftazidime/avibactam, amikacin and colistin also displayed potent activity against the full collection of *P. aeruginosa*, each inhibiting >95% of the isolates.

**Figure 1.**
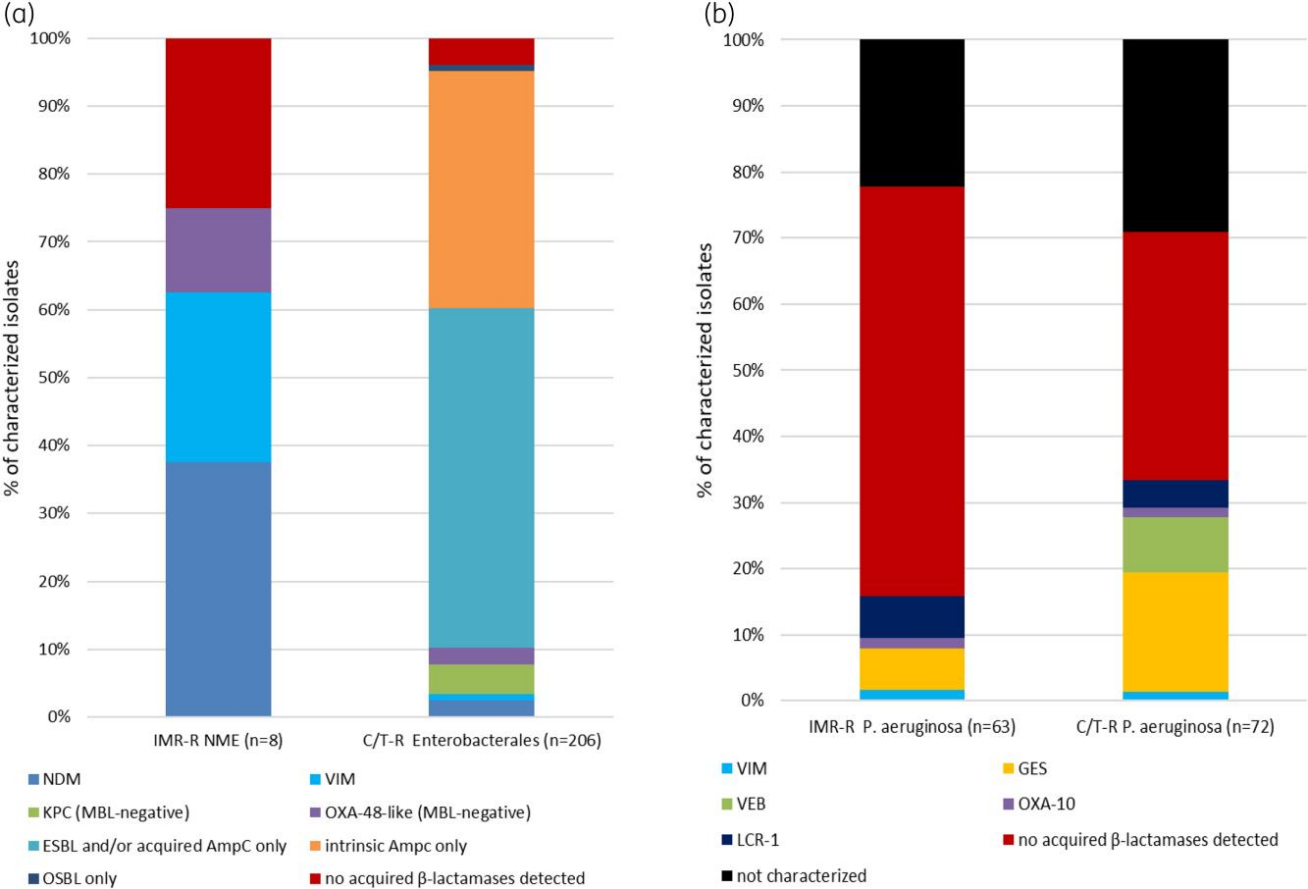
β-Lactamase content among imipenem/relebactam and ceftolozane/tazobactam resistant NME/Enterobacterales (a) and *P. aeruginosa* (b) collected in Israel, 2018–22. C/T, ceftolozane/tazobactam; ESBL, extended-spectrum β-lactamase; IMR, imipenem/relebactam; MBL, metallo-β-lactamase; NME, non-*Morganellaceae* Enterobacterales; OSBL, original spectrum β-lactamase.

Figure 1 illustrates the acquired β-lactamase carriage among imipenem/relebactam- and ceftolozane/tazobactam-resistant NME and Enterobacterales, as well as that of imipenem/relebactam- and ceftolozane/tazobactam-resistant *P. aeruginosa.* Five of the eight imipenem/relebactam-resistant NME carried an MBL (NDM, *n* = 3; VIM, *n* = 2), while one isolate harboured OXA-48. No acquired β-lactamases were found in the remaining two. Both of these were identified as *E. coli* and may harbour non-β-lactamase-based resistance mechanisms such as efflux, PBP changes and/or permeability mutations accounting for the elevated MICs. Among the 206 ceftolozane/tazobactam-resistant Enterobacterales, half were found to carry an acquired ESBL or AmpC only, while 72/206 (35.0%) were species known to possess intrinsic (chromosomal) AmpCs with no acquired β-lactamases detected. Most of the remaining isolates harboured carbapenemases: NDM, *n* = 5 (2.4%); VIM, *n* = 2 (1.0%); KPC, *n* = 9 (4.4%); and OXA-48-like, *n* = 5 (2.4%), while two isolates carried only OSBLs (i.e. original spectrum β-lactamases, i.e., TEM-1, SHV-1, etc.), and for eight isolates, no acquired β-lactamases could be detected.

Most of the 63 *P. aeruginosa* resistant to imipenem/relebactam did not carry an acquired β-lactamase (61.9%), while 10 isolates did, including GES (*n* = 4, 6.3%), LCR-1 (*n* = 4, 6.3%), OXA-10, (*n* = 1, 1.6%) and VIM (*n* = 1, 1.6%). Fourteen isolates were not characterized. Similarly, no acquired β-lactamases were observed in 37.5% (27/72) of ceftolozane/tazobactam-resistant *P. aeruginosa*. Among those with acquired β-lactamases, GES was most common 13/72 (18.1%), six carried VEB (8.3%), three (4.2%) carried LCR-1, and one each (1.6%) carried VIM and OXA-10. Twenty-one of the C/T-R *P. aeruginosa* were not molecularly characterized. MIC value frequency distributions for both ceftolozane/tazobactam and imipenem/relebactam among all the molecular-characterized Enterobacterales and *P. aeruginosa* stratified by β-lactamase carriage in were provided in Table S2.

## Discussion

Previous reports on antimicrobial resistance in Israel are limited. Often Israel is included as part of broader Middle Eastern or European data sets, making interpretation of the information specific to Israel difficult to ascertain, although, in general, resistance rates tend to be lower than other countries in the region. For example, Sader *et al*.^13^ reported that the percentages of Israeli isolates susceptible to ceftolozane/tazobactam among Enterobacterales (93.3%) and *P. aeruginosa* (98.2%) collected from 2012 to 2018 were higher than that of most surrounding countries. Similarly, an earlier SMART publication showed that 94.5% (*n* = 1126) of *P. aeruginosa* collected in Israel were ceftolozane/tazobactam susceptible, the second highest percentage among countries surveyed in the Middle East/Africa region.^14^ Data generated by the SENTRY surveillance programme in 2013–16 noted a 22.8% MDR rate among Enterobacterales collected in Israel,^15^ nearly identical to that reported here (22.6%), values slightly lower than the 30% found in a recent mini-review,^16^ with the variation likely due to differences in isolate infection sources and patient populations. The current study is unique in that it is the first to report Israel-specific imipenem/relebactam susceptibility data for both Enterobacterales and *P. aeruginosa*, while confirming high rates of susceptibility of *P. aeruginosa* to ceftolozane/tazobactam.

Ceftolozane/tazobactam inhibited 95% of Enterobacterales, including 95% of the putative AmpC/ESBL-positive non-CRE phenotype *E. coli* and 87% of AmpC/ESBL-positive non-CRE phenotype *K. pneumoniae*. The activity of ceftolozane/tazobactam against *P. aeruginosa* (94% susceptible) surpassed that of standard first-line empiric agents (piperacillin/tazobactam, cefepime and ceftazidime) and a commonly used carbapenem, meropenem. Amikacin and colistin each inhibited >95% of the *P. aeruginosa*; however, despite their excellent *in vitro* activity, it is important to note the practical limitations associated with these agents in treating Gram-negative infections. EUCAST only publishes bracketed colistin and amikacin (systemic infections) MIC breakpoints with a warning against the use of these agents without additional therapeutic measures. Similarly, CLSI does not publish susceptible MIC breakpoints for colistin against any Gram-negative pathogen.

Imipenem/relebactam inhibited over 99% of NME and 100% of the putative AmpC/ESBL-positive non-CRE phenotype *E. coli* and *K. pneumoniae*. Imipenem/relebactam was specifically developed to retain activity against isolates carrying KPCs and generally retains *in vitro* activity against those without MBL-type carbapenemases and has variable activity against organisms with OXA-48-like enzymes. Geographic differences in β-lactamase prevalence and other resistance mechanisms affect the *in vitro* activities of all currently available β-lactams and β-lactam/β-lactamase inhibitor combinations, including ceftolozane/tazobactam and imipenem/relebactam. In the current study, we observed low numbers of MBLs, KPC, OXA-48-like and GES carbapenemases in both Enterobacterales and *P. aeruginosa* (Figure 1). Therefore, mechanisms of carbapenem resistance other than β-lactamases (e.g. OprD mutations in combination with AmpC hyperproduction in *P. aeruginosa*) likely predominated in the isolates we studied.

In conclusion, recent clinical isolates of Enterobacterales and *P. aeruginosa* collected in Israel from 2018 to 2022 showed high susceptibility (≥93%) to ceftolozane/tazobactam and imipenem/relebactam. If the *in vitro* data described here translate into successful clinical results, ceftolozane/tazobactam and imipenem/relebactam could be vital treatment options for patients in Israel with infections caused by Gram-negative pathogens, including antimicrobial-non-susceptible and -resistant Enterobacterales *and P. aeruginosa*.

## Supplementary Material

dlae150_Supplementary_Data

## Transparency declarations

M.G.W. and D.F.S. are employees of IHMA, which receives funding from Merck Sharp & Dohme LLC, a subsidiary of Merck & Co., Inc., Rahway, NJ, USA for the SMART surveillance program. C.A.D. is a consultant to IHMA. F.S., I.A., K.Y., and M.R.M. are employees of Merck Sharp & Dohme LLC, a subsidiary of Merck & Co., Inc., Rahway, NJ, USA, and own stock in Merck & Co., Inc., Rahway, NJ, USA. The IHMA authors and C.A.D. do not have personal financial interests in the sponsor of this manuscript (Merck Sharp & Dohme LLC, a subsidiary of Merck & Co., Inc., Rahway, NJ, USA). All authors provided analysis input and have read and approved the final manuscript.

## Supplementary data

Tables S1 and S2 are available as Supplementary data at *JAC-AMR* Online.
