# Supplementary material for: Susceptibility of Gram-negative pathogens collected in Israel to ceftolozane/tazobactam, imipenem/relebactam and comparators: SMART 2018–22
Source: JAC Antimicrob Resist. 2024 Sep 17;6(5):dlae150. doi: 10.1093/jacamr/dlae150 (PMC11406551; doi:10.1093/jacamr/dlae150)
Supplement: dlae150_Supplementary_Data [file dlae150_supplementary_data.docx]

**Supplemental Table S1.** Demographic/clinical characteristics of Gram-negative bacilli collected in Israel in 2018-2022

|  | ***n* (% of total)** | | |
| --- | --- | --- | --- |
| **Demographic/clinical characteristics** | **Enterobacterales^a^**  **(*n*=4,220)** | | ***P. aeruginosa* (*n*=1182)** |
| Year of collection |  | |  |
| 2018 | 1045 (24.8%) | | 301 (25.5%) |
| 2019 | 1077 (25.5%) | | 292 (24.7%) |
| 2020 | 827 (19.6%) | | 235 (19.9%) |
| 2021 | 653 (15.5%) | | 181 (15.3%) |
| 2022 | 618 (14.6%) | | 173 (14.6%) |
| Infection source |  | |  |
| Bloodstream | 1004 (23.8%) | | 100 (8.5%) |
| Intraabdominal | 898 (21.3%) | | 130 (11.0%) |
| Lower respiratory tract | 1136 (26.9%) | | 845 (71.5%) |
| Urinary tract | 1147 (27.2%) | | 99 (8.4%) |
| Not specified | 35 (0.8%) | | 8 (0.7%) |
| Patient location |  | |  |
| ICU | 725 (17.2%) | | 299 (25.3%) |
| Non-ICU | 3281 (77.7%) | | 775 (65.6%) |
| Not specified | 214 (5.1%) | | 108 (9.1%) |
| Length of hospitalization at time of specimen collection | |  |  |
| <48 hours | 1,937 (45.9%) | | 405 (34.3%) |
| ≥48 hours | 1835 (43.5%) | | 620 (52.5%) |
| Not specified | 448 (10.6%) | | 157 (13.3%) |

^a^Includes: *Citrobacter amalonaticus (5), Citrobacter braakii (3), Citrobacter farmeri (4), Citrobacter freundii (49), Citrobacter koseri (118), Citrobacter sedlakii (3), Citrobacter sp (1), Enterobacter asburiae (2), Enterobacter bugandensis (23), Enterobacter cloacae (133), Enterobacter cloacae* complex *(14), Enterobacter sp (53), Enterobacter xiangfangensis (1), Escherichia coli (1883), Hafnia alvei (1), Klebsiella aerogenes (123), Klebsiella oxytoca (100), Klebsiella pneumoniae (923), Klebsiella sp (12), Klebsiella variicola (22), Leclercia adecarboxylata (1), Morganella morganii (66), Pantoea agglomerans (1), Pantoea sp (1), Pluralibacter gergoviae (3), Proteus hauseri (7), Proteus mirabilis (351), Proteus penneri (4), Proteus sp (6), Proteus vulgaris (4), Providencia rettgeri (6), Providencia stuartii (53), Raoultella ornithinolytica (1), Raoultella sp (2), Salmonella sp (10), Serratia liquefaciens (5), Serratia marcescens (167), Serratia odorifera (1), Serratia rubidaea (1), Serratia sp (53), Serratia ureilytica (4).*

**Supplemental Table S2.** Ceftolozane/tazobactam and imipenem/relebactam MIC distributions for Enterobacterales and *P. aeruginosa* stratified by β-lactamase content

| **Organism group** | **β-lactamase** | **Agent** | **MIC, mg/L; number of isolates (cumulative % of isolates inhibited at MIC)** | | | | | | | |  |
| --- | --- | --- | --- | --- | --- | --- | --- | --- | --- | --- | --- |
|  |  |  | **≤0.12** | **0.25** | **0.5** | **1** | **2** | **4** | **8** | **≥16** | **Total** |
| Enterobacterales | MBL^a^ | C/T |  |  |  |  |  |  |  | 7 (100) | 7 |
|  |  | IMR |  |  |  |  | 1 (14.3) |  | 4 (71.4) | 2 (100) | 7 |
|  | KPC ^b^ | C/T |  |  |  |  |  |  | 2 (22.2) | 7 (100) | 9 |
|  |  | IMR | 2 (22.2) | 6 (88.9) |  |  | 1 (100) |  |  |  | 9 |
|  | OXA-48-like ^c^ | C/T |  |  |  |  |  |  | 2 (40) | 3 (100) | 5 |
|  |  | IMR | 2 (40) | 1 (60) |  | 1 (80) |  |  |  | 1 (100) | 5 |
|  | Acquired AmpC ^d^ | C/T |  |  | 3 (14.3) | 1 (19.0) |  | 8 (57.1) | 3 (71.4) | 6 (100) | 21 |
|  |  | IMR | 8 (38.1) | 3 (52.4) | 3 (66.7) | 4 (85.7) | 1 (90.5) | 2 (100) |  |  | 21 |
|  | Class A ESBL ^e^ | C/T |  |  |  |  | 1 (1.1) | 28 (33.3) | 34 (72.4) | 24 (100) | 87 |
|  |  | IMR | 58 (66.7) | 19 (88.5) | 4 (93.1) | 2 (95.4) | 4 (100) |  |  |  | 87 |
| *P. aeruginosa* | MBL ^f^ | C/T |  |  |  |  |  |  |  | 1 (100) | 1 |
|  |  | IMR |  |  |  |  |  |  |  | 1 (100) | 1 |
|  | Class A ESBL ^g^ | C/T |  |  |  |  |  | 2 (9.5) | 1 (14.3) | 18 (100) | 21 |
|  |  | IMR | 1 (4.8) | 1 (9.5) | 7 (42.9) | 3 (57.1) | 5 (81) | 2 (90.5) | 1 (95.2) | 1 (100) | 21 |
|  | Class D ^h^ | C/T |  |  |  | 2 (33.3) |  |  | 1 (50) | 3 (100) | 6 |
|  |  | IMR |  |  |  |  | 1 (16.7) | 2 (50) | 2 (83.3) | 1 (100) | 6 |

C/T = ceftolozane/tazobactam; IMR = imipenem/relebactam.

^a^ includes isolates carrying NDM-1 (n=5) and VIM-4 (n=2)

^b^ includes isolates carrying KPC-2 (n=3) and KPC-3 (n=6)

^c^ includes isolates carrying OXA-48 (n=4) and OXA-244 (n=1)

^d^ includes isolates carrying CMY (n=8), DHA (n=12) and MIR (n=1)

^e^ includes isolates carrying CTX-M (n=81), SHV-ESBL (n=3), GES-ESBL (n=1), VEB (n=1). One isolate co-carried CTX-M and SHV-ESBL.

^f^ includes one isolate carrying VIM-2

^g^ includes isolates carrying VEB (n=6) and GES-ESBL (n=15)

^h^ includes isolates carrying LCR-1 (n=5) and OXA-10 (n=1)
